# Physiological serum total bilirubin concentrations were inversely associated with diabetic peripheral neuropathy in Chinese patients with type 2 diabetes: a cross-sectional study

**DOI:** 10.1186/s13098-019-0498-7

**Published:** 2019-12-02

**Authors:** Pijun Yan, Zhihong Zhang, Ying Miao, Yong Xu, Jianhua Zhu, Qin Wan

**Affiliations:** 1 Department of Endocrinology, The Affiliated Hospital of Southwest Medical University, Luzhou, 646000 Sichuan China; 2 Department of General Medicine, The Affiliated Hospital of Southwest Medical University, Luzhou, 646000 Sichuan China

**Keywords:** Total bilirubin, Diabetic peripheral neuropathy, Inflammation, Vasculopathy

## Abstract

**Background:**

Although bilirubin has been generally regarded as a waste with potential neurotoxicity at high levels, a few clinical studies suggest a potential protective role of physiological serum total bilirubin (TBIL) concentrations in diabetic peripheral neuropathy (DPN). However, the pathological mechanisms underlying the relationship remain poorly understood. The objective of this study was to explore the relationship between serum TBIL and DPN, and clinical and laboratory parameters.

**Methods:**

Serum TBIL was measured in 1342 patients with type 2 diabetes mellitus (T2DM). The relationship between TBIL and DPN and other parameters was analyzed.

**Results:**

Serum TBIL levels were significantly lower in T2DM patients with DPN, and were independently and negatively associated with vibration perception thresholds (VPT) (*P *< 0.01 or *P *< 0.05). Moreover, serum TBIL was negatively associated with neutrophil and white blood cell counts, fibrinogen, and the prevalence of hypertension, diabetic foot ulceration, peripheral arterial disease, diabetic nephropathy and diabetic retinopathy (*P *< 0.01 or *P *< 0.05). Additionally, serum TBIL was an independent decisive factor for the presence of DPN after multivariate adjustment. Compared to the highest quartile of TBIL, the lower quartiles were associated with a significantly increased risk of DPN (*P *< 0.01). Last but most importantly, the analysis of receiver operating characteristic curves revealed that the best cutoff value for serum TBIL to predict DPN was 10.75 μmol/L (sensitivity 54.6% and specificity 62.9%).

**Conclusions:**

These findings suggest that lower physiological serum TBIL may be associated with the presence of DPN due to its decreased anti-inflammatory and vascular protective effects.

## Background

Diabetic peripheral neuropathy (DPN), one of the most common long-term complications of type 2 diabetes mellitus (T2DM) with an estimated lifetime prevalence of more than 50%, is characterized by sensory and motor neuron damage [1, 2]. DPN is a major risk factor for foot ulcers and even amputations, resulting in serious health consequences and a heavy economic burden. Although persistent diabetes and hyperglycemia play a central role in the development of DPN, strict glycaemic control cannot eliminate the risk of DPN, suggesting a multifactorial origin in its pathogenesis. Therefore, there is an urgent need to early identify and appropriately control novel modifiable risk factors that contribute to DPN for preventing and treating DPN at an early stage.

Bilirubin, as the end product of heme catabolism in mammals, has been generally regarded merely as a waste with potential neurotoxicity at high levels leading to neurologic dysfunctions and mental disorders [2, 3]. Consistently, Hendrickse et al. reported that serum bilirubin negatively correlated with sural and peroneal nerve conduction velocity, and the severity of autonomic dysfunction (as reflected by the number of abnormal cardiovascular reflex tests) in a group of patients with primary biliary cirrhosis [4]. In contrast, numerous recent studies have reported that bilirubin acts as a potent cellular anti-apoptotic, antioxidant, anti-inflammatory, and immunoregulatory agent at normal to mildly elevated levels, pointing to a cytoprotective and neuroprotective effect of bilirubin [5–7]. Consistent with this notion, serum bilirubin levels have been reported to be inversely associated with the prevalence and the severity of cardiovascular autonomic neuropathy (CAN) [5] and the presence and progression of DPN including distal symmetrical polyneuropathy (DSPN) [2, 8], and positively associated with hands and feet electrochemical skin conductance levels for reflecting sudomotor function [9], and corneal nerve fibers morphology for reflecting small nerve fibers (unmyelinated C-fibers) dysfunction [10] in patients with T2DM. However, the pathological mechanisms underlying the relationship between physiological serum TBIL concentrations and DPN has not been fully understood.

Therefore, the cross-sectional study was conducted to investigate the relationship between physiological serum TBIL concentrations and DPN in a Chinese population with T2DM. Moreover, the potential associations among serum TBIL and liver enzymes, metabolic parameters, inflammatory markers, and other diabetic micro- and macrovascular complications were also evaluated.

## Methods

### Study population

3514 patients with T2DM, consecutively attending the inpatient department of Endocrinology at the Affiliated Hospital of Southwest Medical University, Luzhou, China, from August 2012 to September 2015, were initially recruited. All patients had a diagnosis of T2DM according to an oral glucose tolerance test and the American Diabetes Association criteria in 1997. Subject inclusion criteria were as follows: (1) confirmed or newly diagnosed T2DM; (2) normal liver function tests [defined as TBIL < 22.0 μmol/L, conjugated bilirubin < 7 μmol/L, alanine aminotransferase (ALT) < 50U/L, and aspartate aminotransferase (AST) < 40 U/L]; (3) age between 24 and 89 years; and (4) long-term residence (≥ 5 years) in the Sichuan province. Excluded were patients with type 1 diabetes and other endocrine disorders such as thyroid disease, and parathyroid disease, acute complications of diabetes such as ketoacidosis or hyperosmolar state, severe respiratory and cerebrovascular disease, history of seizures or epilepsy, arrhythmia including atrial fibrillation, heart failure, renal failure, chronic liver disease including viral hepatitis B or C, liver cirrhosis, inflammatory or autoimmune diseases, acute infectious disease, cancer, alcoholism, acute or chronic blood loss, hemolysis, or red blood cell transfusion, pregnancy or lactation, current treatment with immunosuppressive medications, anti-inflammatory medications and analgesics, systemic corticosteroids, and, prior history of knee or back surgery, any other etiological cause of peripheral neuropathy, and patients with use of any drugs known to interfere with oxidant/antioxidant system, peripheral nerve function and sympathetic system such as beta blockers. After the exclusions, a total of 1342 participants (638 men and 704 women) were included in the final analysis, and subsequently divided into two groups: diabetic peripheral neuropathy (DPN group) and no diabetic peripheral neuropathy (no DPN group) according to previous published work [11]. The study was performed in accordance with the ethical guidelines of the 1975 Declaration of Helsinki and was reviewed and approved by the human research ethics committee of the Affiliated Hospital of Southwest Medical University, and informed consent was obtained from all T2DM patients prior to participation.

### Anthropometric and biochemical measurements

Demographic information, including his/her diabetic duration, medical history, lifestyle factors, alcohol consumption, cigarette smoking, and history of hypertension, stroke, coronary heart disease (CHD), and the presence of diabetic complications, including diabetic foot ulceration (DFU), peripheral arterial disease (PAD), diabetic retinopathy (DR), and diabetic nephropathy (DN), was collected by a standardized questionnaire, and a comprehensive physical examination were performed according to standard procedures in all participants. Body weight and height were taken using standard protocols with the subjects in light clothing and without shoes. Body mass index (BMI) was calculated with the following formula: BMI = body weight (kg)/height (m)^2^. Systolic and diastolic blood pressures were measured on the right arm using a standard mercury sphygmomanometer. Pulse pressure (PP) and mean arterial pressure (MAP) were calculated, as described by Xu et al. [12].

Blood samples were collected from study participants in the morning either after at least 8 h of fasting. Blood samples were collected by centrifugation at 3500 rpm for 10 min at 4 °C and stored at − 80 °C until analytical processing. Fasting blood glucose (FBG) and glycated hemoglobin A1C (HbA1c) levels were measured by the glucose-oxidase method and anion exchange high performance liquid chromatography, respectively (arkray ELUENT 80A, Japan). Serum levels of AST, ALT, lipid profiles, including total cholesterol (TC), triglyceride (TG), high density lipoprotein cholesterol (HDL-C), low-density lipoprotein cholesterol (LDL-C), apolipoprotein A (apoA), apolipoprotein B (apoB), and creatinine (Crea) were analyzed enzymatically using a 7060 full-automatic biochemical analyzer (Hitachi, Tokyo, Japan) at a certified laboratory. ApoB/ApoA equals the ration between ApoB and ApoA. Serum levels of total, direct and indirect bilirubin were measured by the oxidation method. The normal range of serum total bilirubin levels used in the study was 5–22 μmol/L. The peripheral leukocyte count (white blood cell, WBC) count, neutrophil and lymphocyte counts were determined using an automated blood cell counter (Mindray BC-6800, Shenzhen, China), according to the manufacturer’s instructions. Neutrophil to lymphocyte ratio (NLR) was calculated as neutrophil count divided by lymphocyte count. The coagulation function tests, including prothrombin time (PT), and activated partial thromboplastin time (APTT), international normalized ration (INR), fibrinogen, were determined coagulation method. The estimated glomerular filtration rate (eGFR), a measure of renal function, was estimated using Chronic Kidney Disease Epidemiology Collaboration (CKD-EPI) equations modified by a Japanese coefficient [13]. Urinary microalbumin and creatinine were measured, and the urinary albumin-to-creatinine ratio (ACR; mg/g creatinine) was calculated, as described by us [14]. DN was defined as the absence of signs or symptoms of other primary causes of kidney damage, the presence of albuminuria (ACR ≥ 30 mg/g Crea) or an eGFR < 60 mL/min/1.73 m^2^ [8, 15].

### Foot examination

All T2DM patients were asked whether they had a history for claudication, and underwent a comprehensive foot examination including assessment of foot pulses (dorsalis pedis and tibialis posterior arteries). Then a diagnostic ankle–brachial index (ABI) was measured. Leg-specific ABI was calculated by dividing the higher systolic blood pressure (SBP) in the posterior tibial or dorsalis pedis by the higher of the right or left brachial SBP. Patients were diagnosed as having PAD, as described by Levey and us [14, 15].

VPT was assessed at the metatarsophalangeal joint dig I using a neurothesiometer (Bio-Thesiometer; Bio-Medical Instrument Co., Newbury, OH, USA) according to previously published methods. VPT was measured by the same person with considerable experience in this procedure on every occasion. Sensibility to touch was tested using 10-g Semmes–Weinstein monofilament (SWM) at four points on each foot. The same experienced physician performed all the above measurements in a quiet, warm, relaxed environment. DPN was defined as VPT ≥ 25 V and/or inability to feel the monofilament [11].

DFU is defined as “ulceration of the foot (distally from the ankle and including the ankle) associated with neuropathy and different grades of ischemia and infection” according to the World health Organization [16].

### Other classifications and definitions

The presence of DR was assessed by high-quality fundus photographs and an ophthalmologist, as described by us [14].

Hypertension was defined as a blood pressure ≥ 140/90 mmHg at examination or presence of antihypertensive treatment [6, 11].

CHD was defined as ≥ 1 event of myocardial infarction or coronary revascularization procedure [17].

Stroke was considered present if diagnosed according to previous medical records or if a pathological finding by computed tomography (CT) and brain magnetic resonance imaging (MRI) of the brain [18].

### Statistical analysis

All analyses were performed with the Statistical Package for Social Sciences version 20.0 (SPSS, Chicago, IL). All data were first analyzed for normality of distribution using the Kolmogorov–Smirnov test of normality, and homogeneity of variance using the Levene homogeneity of variance test. Data are expressed as mean ± standard deviation (SD) for continuous variables or number (percentages) for categorical variables.

Two-group comparisons were performed with *χ*^2^ test for categorical variables or Student’s *t* test for normally distributed continuous variables or Mann–Whitney U test for nonparametric distributed continuous variables. Comparisons among more than two groups were performed with one-way analysis of variance (ANOVA) (continuous variables with normally distribution and homogeneity of variance), or the Kruskal–Wallis test (covariates with nonparametric distribution and/or variance uneven). Comparison of serum TBIL levels between groups with and without DNP was further performed with analysis of covariance (ANCOVA) adjusting for age, sex, BMI, and diabetic duration because of the difference of baseline characteristics such as age, sex, BMI, and diabetic duration. The association between serum TBIL and other variables was investigated by Pearson or Spearman bivariate correlation analysis; the partial correlation coefficient was used to control for the effects of age, sex, BMI, and diabetic duration. Multiple stepwise linear regression analysis was subsequently performed to examine the independent variables associated with **s**erum TBIL in T2DM patients. The univariate and multivariable logistic regression analyses were also performed to determine the association of serum TBIL and other variables with risk of DPN. Odds ratios (OR) and 95% confidence intervals (CI) were estimated. We then categorized patients into 4 quartile groups by the TBIL level. Binary logistic regression analyses were conducted to investigate the association between quartiles of TBIL and DPN. The highest quartile (Q1) served as the reference group, and OR and 95% CI were estimated. Possible dose–response relationships between TBIL and DPN were examined by the trend test. Further, we compared the prevalence of DPN across the 4 TBIL quartile categories in all T2DM patients using ANOVA. Receiver operating characteristic (ROC) curve analysis was performed to determine the optimal cut-off point of serum TBIL levels for the diagnosis of DPN.

In all statistical tests, a *P*-value of < 0.05 was considered to be statistically significant (two sided).

## Results

### Circulating TBIL levels and other clinical characteristics of studied population

A total of 1342 patients with T2DM (mean age, 60.16 ± 10.98 years; male/female, 638/704; and mean diabetes duration, 7.83 ± 6.49 years) were finally enrolled in this study. The anthropometric, biochemical and clinical parameters of study population are shown in Table 1. When compared with those without, T2DM patients with DPN had significantly more men, older age, longer diabetic duration, larger proportions of hypertension, CHD, stroke, DFU, PAD, DN, and DR, higher levels of blood pressure, glycemic control, reflected by FBG and HbA1c, ApoB/ApoA, neutrophil count, WBC, NLR, coagulation index, urinary ACR, Crea and VPT values, and lower levels of BMI, DBP, TC, TG, ApoA, TBIL, DBIL, IBIL, eGFR, liver enzymes, lymphocyte count, and ABI (*P* < 0.01 or *P* < 0.05).

**Table 1:** Circulating TBIL levels and other clinical characteristics between T2DM patients with and without DPN

Variables	No DPN	DPN	*P*-value
	(n = 1129)	(n = 213)	
Male/female (n)	522/607	116/97	0.027
Age (years)	58.94 ± 10.74	66.65 ± 9.90	0.000
BMI (kg/m^2^)	24.17 ± 3.57	23.42 ± 3.99	0.003
Diabetic duration (years)	7.30 ± 6.16	10.61 ± 7.46	0.000
SBP (mmHg)	131.96 ± 20.38	135.63 ± 22.63	0.016
DBP (mmHg)	71.95 ± 11.93	70.04 ± 12.88	0.034
PP(mmHg)	60.03 ± 18.37	65.59 ± 19.32	0.000
MAP(mmHg)	91.96 ± 12.60	91.91 ± 14.09	0.958
FBG (mmol/L)	10.62 ± 5.15	11.53 ± 5.45	0.005
HbA1c (%)	9.38 ± 2.49	10.12 ± 2.63	0.000
TC (mmol/L)	4.91 ± 1.34	4.67 ± 1.29	0.011
TG (mmol/L)	2.37 ± 2.44	1.87 ± 1.38	0.002
HDL-C (mmol/L)	1.18 ± 0.35	1.18 ± 0.37	0.445
LDL-C (mmol/L)	2.81 ± 1.01	2.76 ± 0.97	0.503
ApoA (g/L)	1.35 ± 0.29	1.27 ± 0.33	0.000
ApoB (g/L)	0.91 ± 0.28	0.91 ± 0.36	0.591
ApoB/ApoA	0.70 ± 0.25	0.76 ± 0.36	0.026
TBIL (μmol/L)	11.63 ± 3.87	10.02 ± 3.61	0.000
TBIL (μmol/L)^a^	11.62 ± 3.86	9.96 ± 3.60	0.000
DBIL (μmol/L)	4.08 ± 1.46	3.70 ± 1.39	0.000
IBIL (μmol/L)	7.55 ± 2.92	6.32 ± 2.65	0.000
ALT (U/L)	19.40 ± 9.00	15.65 ± 8.02	0.000
AST (U/L)	18.93 ± 6.09	17.37 ± 6.14	0.000
Neutrophil count (* 10^9^/L)	4.42 ± 2.01	5.34 ± 2.85	0.000
Lymphocyte count (* 10^9^/L)	1.68 ± 0.63	1.46 ± 0.57	0.000
NLR	3.10 ± 2.58	4.40 ± 4.00	0.000
WBC (* 10^9^/L)	6.65 ± 2.18	7.41 ± 3.03	0.000
PT (s)	12.44 ± 1.15	13.12 ± 2.00	0.000
APTT (s)	31.36 ± 5.93	33.26 ± 8.60	0.007
INR	1.02 ± 0.08	1.07 ± 0.19	0.001
Fibrinogen (g/L)	3.55 ± 1.23	4.43 ± 1.59	0.000
ACR (mg/g)	212.59 ± 24.67	445.13 ± 73.42	0.000
eGFR (mL/min/1.73 m^2^)	93.80 ± 25.11	77.72 ± 27.45	0.000
Crea (μmol/L)	71.05 ± 45.82	88.25 ± 50.21	0.000
VPT (V)	13.03 ± 4.72	36.67 ± 8.88	0.000
ABI	1.03 ± 0.13	0.95 ± 0.24	0.000
Hypertension (n, %)	565 (50.04)	135 (63.38)	0.000
CHD (n, %)	87 (7.71)	37 (17.37)	0.000
Stroke (n, %)	213 (18.87)	71 (33.33)	0.000
DFU (n, %)	54 (4.78)	47 (22.07)	0.000
PAD (n, %)	80 (7.09)	57 (26.76)	0.000
DN (n, %)	385 (34.10)	113 (53.05)	0.000
DR (n, %)	128 (11.34)	47 (22.07)	0.000

Data are mean ± SD

*SD* standard deviation, *DPN* diabetic peripheral neuropathy, *BMI* body mass index, *SBP* systolic blood pressure, *DBP* diastolic blood pressure, *PP* pulse pressure, *MAP* mean arterial pressure, *FBG* fasting blood glucose, *HbA1c* glycated hemoglobin A1c, *TC* total cholesterol, *TG* triglyceride, *HDL-C* high-density lipoprotein cholesterol, *LDL-C* low-density lipoprotein cholesterol, *apoA* apolipoprotein A, *apoB* apolipoprotein B, *ApoB/ApoA* apolipoprotein B–apolipoprotein A ratio, *TBIL* total bilirubin, *DBIL* direct bilirubin, *IBIL* indirect bilirubin, *ALT* alanine aminotransferase, *AST* aspartate aminotransferase, *NLR* neutrophil to lymphocyte ratio, *WBC* white blood cell, *PT* prothrombin time, *APTT* activated partial thromboplastin time, *INR* international normalized ration, *ACR* albumin- to-creatinine ratio, *eGFR* estimated glomerular filtration rate, *Crea* creatinine, *VPT* vibration perception threshold, *ABI* ankle–brachial index, *CHD* coronary heart disease, *DFU* diabetic foot ulceration, *PAD* peripheral arterial disease, *DN* diabetic nephropathy, *DR* diabetic retinopathy

^a^Adjustment for age, sex, BMI, and diabetic duration performed with analysis of covariance (ANCOVA) (dependent variable: serum TBIL level, fixed factor: group, and covariates: age, sex, BMI, and diabetic duration)

### Association of serum TBIL with anthropometric, biochemical and clinical parameters in study subjects

Next, we analyzed the relationship of serum TBIL with various other parameters by using simple correlations. In all T2DM patients, serum TBIL levels were positively associated with DBP, TC, HDL-C, ApoA, LDL-C, DBIL, IBIL, liver enzymes, and eGFR, and negatively with age, sex, diabetic duration, PP, TG, ApoB/ApoA, neutrophil and WBC counts, fibrinogen, urinary ACR, Crea, VPT values, and the prevalence of hypertension, DFU, PAD, DPN, DN and DR (*P *< 0.01 or *P *< 0.05; Table 2). With adjustment for age, sex, BMI, and diabetic duration, serum TBIL levels correlated significantly and positively with HDL-C, ApoA, DBIL, IBIL, and liver enzymes (all *P *< 0.01). A negative correlation of serum TBIL with diabetic duration, PP, TG, ApoB/ApoA, neutrophil and WBC counts, NLR, fibrinogen, urinary ACR, VPT values, and the prevalence of hypertension, DFU, DPN, DN and DR was also observed (*P *< 0.01 or *P *< 0.05). Furthermore, we performed multiple stepwise regressions models to identify the main determinants of serum TBIL in people with T2DM. The results showed that sex, HDL-C, ALT, PT, INR, fibrinogen, urinary ACR, and VPT values were independent factors determining serum TBIL (Table 2). The multiple regression equation was: Y_TBIL_ = 10.913 − 1.141X_urinary_ _ACR_ +0.046X_ALT_ − 0.357X_fibrinogen_ + 0.823X_PT_ − 0.032X_VPT_ − 1.012X_sex_ + 1.557X_HDL-C_ − 0.357X_fibrinogen_ + 7.203 X_INR_ (*P *< 0.01 or *P *< 0.05; Table 2).

**Table 2:** Linear correlation and multiple regression analysis of variables associated with serum TBIL in the entire population studied

Variable	Simple				Multiple		
	r	*P*-value	Adjusted r	Adjusted *P*-value	β	Standardized β	*P*-value
Age	− 0.071	0.010					
Sex	− 0.095	0.000			− 1.012	− 0.128	0.004
BMI	0.047	0.091					
Diabetic duration	− 0.132	0.000					
SBP	− 0.045	0.101	− 0.084	0.067			
DBP	0.070	0.010	0.041	0.370			
PP	− 0.084	0.002	− 0.131	0.004			
MAP	0.021	0.435	− 0.017	0.712			
FBG	0.019	0.489	− 0.049	0.281			
HbA1c	− 0.011	0.698	− 0.069	0.129			
TC	0.095	0.001	0.041	0.365			
TG	− 0.066	0.016	− 0.095	0.038			
HDL-C	0.172	0.000	0.175	0.000	1.557	0.140	0.002
LDL-C	0.075	0.007	0.014	0.753			
ApoA	0.177	0.000	0.182	0.000			
ApoB	0.039	0.161	− 0.015	0.738			
ApoB/ApoA	− 0.068	0.014	− 0.107	0.019			
DBIL	0.779	0.000	0.770	0.000			
IBIL	0.951	0.000	0.947	0.000			
ALT	0.163	0.000	0.125	0.006	0.046	0.101	0.019
AST	0.123	0.000	0.118	0.009			
Neutrophil count	− 0.074	0.008	− 0.127	0.005			
Lymphocyte count	− 0.045	0.107	0.013	0.785			
NLR	− 0.019	0.506	− 0.120	0.009			
WBC count	− 0.102	0.000	− 0.122	0.008			
PT	0.046	0.237	0.032	0.484	0.823	0.248	0.001
APTT	0.014	0.716	0.038	0.408			
INR	0.012	0.766	− 0.024	0.599	− 7.203	− 0.152	0.037
Fibrinogen	− 0.165	0.000	− 0.206	0.000	− 0.357	− 0.119	0.010
ACR	− 0.227	0.000	− 0.167	0.000	− 1.141	− 0.216	0.000
eGFR	0.166	0.000	0.077	0.093			
Crea	− 0.112	0.000	− 0.075	0.099			
VPT	− 0.165	0.000	− 0.144	0.002	− 0.032	− 0.089	0.047
ABI	0.038	0.166	0.041	0.368			
Hypertension	− 0.108	0.000	− 0.098	0.000			
CHD	0.023	0.393	0.054	0.052			
Stroke	− 0.042	0.126	− 0.008	0.768			
DFU	− 0.126	0.000	− 0.117	0.000			
PAD	− 0.058	0.033	− 0.025	0.364			
DPN	− 0.149	0.000	− 0.139	0.000			
DN	− 0.144	0.000	− 0.130	0.000			
DR	− 0.145	0.000	− 0.119	0.000			

In multiple linear stepwise regression analysis, values included for analysis in T2DM patients were sex, age, BMI, diabetic duration, blood pressure (SBP, DBP, PP, and MAP), glycemic control (FBG and HbA1C), Lipid profiles (TG, TC, HDL-C, LDL-C, ApoB, ApoB, and ApoB/ApoA), liver function (ALT and AST), inflammatory markers (neutrophil, lymphocyte, NLR, and WBC counts), coagulation function (PT, APTT, INR, and *fibrinogen*), renal function (urinary ACR, eGFR, and Crea), VPT values, and ABI

### Multivariable-adjusted ORs for the association of serum TBIL concentration with increased presence of DPN in study subjects

To assess whether serum TBIL concentrations can decrease the risk of development of DPN, univariate and multivariate logistic regression analysis was mapped. As shown in Table 3, univariate logistic regression analysis revealed that sex, BMI, TC, TG, ApoA, TBIL, liver enzymes, lymphocyte count, and eGFR were negative predictors of the presence of DPN, and age, diabetic duration, PP, glycemic control, ApoB/ApoA, neutrophil and WBC counts, NLR, coagulation index, Crea, the prevalence of hypertension, CHD, stroke, DFU, PAD, DN and DR were positive predictors of the presence of DPN. Importantly, in multivariate logistic regression analysis, serum TBIL level was an independent decisive factor for the presence of DPN, even after adjusting for all confounding variables (odds ratio, 0.893; 95% confidence interval, 0.828–0.964; *P* = 0.004)], indicating that there was a 10.7% decrease in the odds of having DPN for each 1 μmol/L increase in TBIL levels.

**Table 3:** Binary logistic regression analyses of variables contributing to DPN in patients with T2DM

Variables	Univariate analysis			Multivariate analysis		
	B	OR (95% CI)	*P*-value	B	OR (95% CI)	*P*-value
Sex	− 0.330	0.719 (0.536–0.965)	0.028			
Age	0.071	1.074 (1.057–1.091)	0.000	0.043	1.044 (1.010–1.079)	0.011
BMI	− 0.059	0.943 (0.902–0.984)	0.008			
Diabetes duration	0.071	1.073 (1.051–1.096)	0.000			
PP	0.015	1.016 (1.008–1.023)	0.000			
MAP	0.000	1.000 (0.988–1.011)	0.953			
FBG	0.031	1.032 (1.005–1.059)	0.019			
HbA1c	0.111	1.117 (1.057–1.181)	0.000			
TC	− 0.147	0.863 (0.765–0.974)	0.017			
TG	− 0.161	0.851 (0.765–0.947)	0.003			
HDL-C	− 0.028	0.972 (0.638–1.482)	0.895			
LDL-C	− 0.046	0.955 (0.821–1.110)	0.547			
ApoA	− 0.984	0.374 (0.220–0.634)	0.000			
ApoB	0.038	1.039 (0.626–1.725)	0.882			
ApoB/ApoA	0.719	2.052 (1.230–3.423)	0.006	3.871	48.012 (1.478–1560)	0.029
TBIL	− 0.119	0.888 (0.851–0.927)	0.000	− 0.113	0.893 (0.828–0.964)	0.004
ALT	− 0.056	0.945 (0.926–0.964)	0.000			
AST	− 0.045	0.956 (0.931–0.981)	0.001			
Neutrophil count	0.153	1.166 (1.099–1.237)	0.000			
Lymphocyte count	− 0.674	0.510 (0.385–0.675)	0.000			
NLR	0.117	1.124 (1.073–1.177)	0.000			
WBC count	0.115	1.122 (1.063–1.185)	0.000			
PT	0.360	1.434 (1.228–1.673)	0.000			
APTT	0.040	1.041 (1.013–1.070)	0.004			
INR	4.457	86.264(11.060–672.807)	0.000			
Fibrinogen	0.431	1.540 (1.346–1.761)	0.000			
eGFR	− 0.022	0.978 (0.973–0.983)	0.000			
Crea	0.006	1.006 (1.003–1.009)	0.000			
Hypertension	0.547	1.728 (1.277–2.337)	0.000			
CHD	0.923	2.518 (1.660–3.819)	0.000			
Stroke	0.766	2.150 (1.559–2.966)	0.000			
DFU	1.729	5.636 (3.689–8.611)	0.000	1.072	2.922 (1.435–5.950)	0.003
PAD	1.567	4.791 (3.279–6.999)	0.000			
DN	0.781	2.184 (1.624–2.936)	0.000			
DR	0.849	2.337 (1.607–3.398)	0.000			

Beta is the standardized coefficient and measures the influence of each variables on DPN; OR is the odds ratio and refers to the risk of DPN

### Association between quartiles of serum TBIL level and the risk of DPN in study subjects

Further, all subjects were separately categorized into four quartile groups (Q1–Q4) according to serum TBIL, and the risk of development of DPN in different TBIL quartiles was assessed. As shown in Table 4, the prevalence of DPN was decreased by 36.8% (95% CI 25.6–46.4%; *P* < 0.01) per standard deviation increase in TBIL. Compared to the lowest quartile of TBIL, the higher quartiles were associated with a significantly decreased risk of DPN in the entire T2DM population (all *P* < 0.01).

**Table 4:** Association between quartiles of serum TBIL and the risk of DPN in the entire T2DM population

TBIL (μmol/L)	DPN	
	Odds ratio (95% CI)	*P*
Per SD increase	0.632 (0.536–0.744)	0.000
Quartiles of TBIL		
Q1 (2.80–8.20)	1 (reference)	
Q2 (8.30–10.90)	0.601 (0.412–0.877)	0.008
Q3 (11.00–14.00)	0.485 (0.324–0.727)	0.000
Q4 (14.10–22.00)	0.317 (0.203–0.495)	0.000
*p* for trend	0.000	
Q4 versus Q1, Q2, Q3	0.464 (0.310–0.695)	0.000

Data are expressed as OR (95% CI) + *P* value, unless stated otherwise

*OR* odds ratio, *CI* confidence interval

### The predictive value of serum TBIL in detecting DPN

**Fig. 1:**
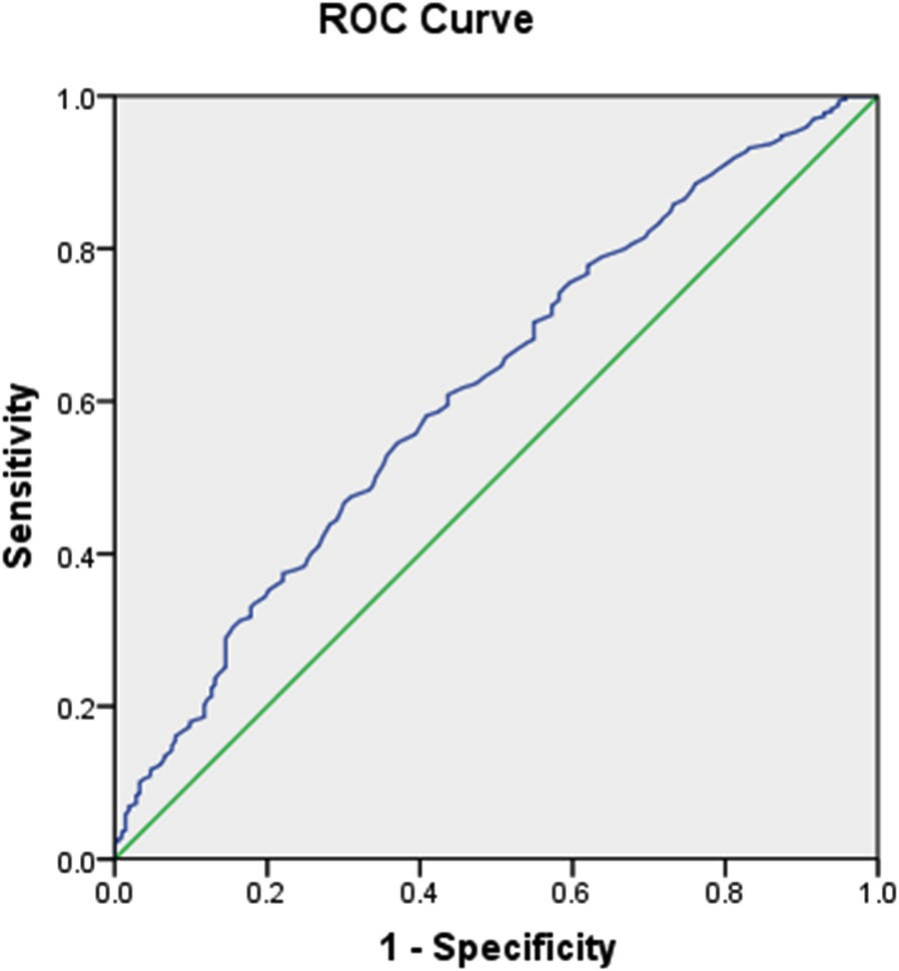
ROC analysis of serum total bilirubin (TBIL) to indicate DPN for T2DM patients. AUC = 0.618; 95% CI 0.577–0.659; *P* = 0.000; identified TBIL cutoff value = 10.75 μmol/L; Youden index = 0.175; sensitivity: 54.6%; specificity: 62.9%

To explore the predictive value of serum TBIL for DPN, we analyzed the ROC curves of serum TBIL. The results revealed that the best cutoff value for serum TBIL to predict DPN was 10.75 μmol/L (sensitivity: 54.6%, specificity: 62.9%, and AUC 0.618) in the entire T2DM population (Fig. 1).

## Discussion

In the present study, we found that serum TBIL levels significantly decreased in T2DM patients with DPN, and were independently and negatively associated with VPT values. We further showed that serum TBIL level was an independent decisive factor for the presence of DPN, even after multivariate adjustment. These findings suggest that serum TBIL may be a useful biomarker of DPN, and lower physiological serum levels of TBIL might be etiologically associated with DPN.

As mentioned earlier, bilirubin, including free bilirubin, albumin–bound bilirubin, conjugated bilirubin and unconjugated bilirubin, is a potent endogenous antioxidant [5]. Serum bilirubin levels are positively correlated with the levels of antioxidative enzyme such as superoxide dismutase, catalase, and glutathione peroxidase, and its antioxidant ability is equal to or more potent than that of α-tocopherol [5]. Bilirubin was capable of efficiently scavenging singlet oxygen, superoxide, peroxynitrite and peroxyl radicals [1, 5, 7]. In addition, bilirubin lowers reactive oxygen species (ROS) and protects against nerve injury by inhibiting the protein kinase C and NAD(P)H oxidase pathway [2, 5, 6, 8]. Moreover, bilirubin might exert a neuroprotective effect by inhibiting formation of advanced glycation end-product which results in nerve fibre damage by the modification of proteins or from impaired nerve blood flow and diminished neurotrophic support [5, 6]. It is well known that oxidative stress is a major contributor in the pathogenesis of DPN [1, 7]. Therefor, these findings suggest that high serum bilirubin at physiological concentrations might exhibit effective antioxidant activities which might play a protective role in the development and progression of DPN. Consistent with this hypothesis, we found that serum bilirubin levels were significantly decreased in T2DM patients with DPN. Moreover, serum TBIL levels were independently and negatively associated with VPT value. VPT measurement may be an useful and reliable method for early screening DPN and reflecting the clinical severity of DPN [1]. These results, together, indicated that there may be a potential mechanistic association between decreased serum TBIL and the development of DPN. Additionally, multivariate logistic regression analysis indicated that serum TBIL was significantly associated with the development of DPN after controlling for all potential confounders. Consistently, the prevalence of DPN was decreased by 36.8% per standard deviation increase in TBIL. Further, the cutoff point of serum TBIL concentration to predict DPN was 10.75 μmol/L. Collectively, these data demonstrate that decreased serum TBIL may be associated with the development of DPN. This hypothesis is strongly supported by some previous clinical studies showing that T2DM patients with DSPN had low levels of serum bilirubin, and serum bilirubin concentrations are inversely associated with the presence and progression of DPN including DSPN in patients with T2DM, independently of classic risk factors and other microvascular complications [2, 8].

Numerous experimental and clinical studies demonstrated that chronic low-grade inflammation as another potential factor in DPN [1, 7, 19]. Experimental findings to date have shown that bilirubin can disturb the expression levels of complement activity, cell adhesion molecules, and suppressing differentiation of T cell [2, 6], and the release of tumour necrosis factor alpha (TNF-α), interleukin (IL)-2 and IL-10, and also down-regulating the expression of major histocompatibility complex class-II expression in macrophages [8]. An inverse association of bilirubin with markers of inflammation, i.e., C-reactive protein (CRP), NLR, and so on were also reported in various diseases [2, 8, 10, 20, 21].

Together, these results suggest that bilirubin possesses potent anti-inflammatory properties in vitro and in vivo. WBC and neutrophil counts, routinely measured classic inflammation markers, are correlated with circulating CRP concentration [14, 22]. Higher level of NLR and lower lymphocyte counts also has recently been Regarded as a dysregulated inflammatory response and increased levels of inflammation [14, 23]. In our study, we found that T2DM patients with DPN had significantly higher levels of neutrophil and WBC counts and NLR, and lower levels of lymphocyte count. Moreover, neutrophil and WBC counts, and NLR were positive predictors of the presence of DPN, whereas lymphocyte count was a negative predictor. Our findings further support an important role of inflammation in the development of DPN. As expected, serum TBIL levels were inversely associated with inflammation markers neutrophil and WBC counts, and NLR.

Based on these findings in our study and previous ones, we extrapolate that the association between circulating bilirubin levels and inflammatory markers may provide new insights into the potential involvement of anti-inflammatory effect of bilirubin in the pathogenesis of DPN in type 2 diabetes.

Numerous experimental and clinical studies have provided solid evidence of an important role for vasculopathy in the pathogenesis of DPN [24, 25]. The pathological basis of diabetic vascular complications including DPN is atherosclerosis. As is well known, atherosclerosis and its consequence, tissue ischaemia and endoneural hypoxia, are characterized by a state of heightened inflammation and oxidative stress [26], while bilirubin possesses antioxidant and anti-inflammatory activities in vitro and vivo, suggesting that decreased serum TBIL may play a protective role against micro- and macrovascular complications of diabetes including DPN. In the present study, we have revealed that T2DM patients with DPN had significantly larger proportions of diabetic microvascular (DR and DN) and macrovascular (hypertension, CHD, stroke, DFU, and PAD) Complications. Moreover, T2DM patients with DPN had significantly higher levels of blood pressure, blood glucose, surrogate markers of atherosclerosis including increased ApoB/ApoA and coagulation index, reduced ApoA, and lower ABI values. Consistently, PP, blood glucose, ApoB/ApoA, coagulation index, the prevalence of other diabetic microvascular and macrovascular complications were positive predictors of the presence of DPN, especially ApoB/ApoA and the prevalence of DFU were independent decisive factors for the presence of DPN. These findings were consistent with previous studies [2, 11, 27, 28], which imply a potential important role of metabolic and vascular disorder associated with atherosclerosis in the development of DPN. In addition, we demonstrated that serum TBIL levels were positively associated with HDL-C, ApoA and eGFR, and negatively with SBP, PP, TG, ApoB/ApoA, urinary ACR, and the prevalence of hypertension, DFU, DN and DR, and HDL-C and urinary ACR were independent factors determining serum TBIL levels, almost consistent with previous studies [5–7, 10]. Surprisingly, we also found that serum TBIL levels were negatively associated with fibrinogen, and PT, INR, and fibrinogen were independent factors determining serum TBIL levels, demonstrating that serum TBIL may negatively regulate coagulation index, and might have anti-thrombotic property. Two previous studies reported a correlation between serum bilirubin and PT and APTT in 1-month-old infants and HIV-infected adults on stable antiretroviral therapy [29, 30]. Recently, Cho et al. reported that high bilirubin concentrations were independently associated with low levels of fibrinogen and plasminogen activator inhibitor-1, respectively, in Korean subjects [31]. More recently, Gligorijević et al. demonstrated that bilirubin and fibrinogen interact at physiological concentrations, bilirubin may exert antioxidant effect on fibrinogen, preventing its carbonylation and aggregation [32]. Our results, together with the previously published studies, support the possibility that bilirubin may confer vascular protective and neuroprotective effects possibly due to its antioxidant, anti-inflammatory, anti-atherosclerosis, and anti-thrombotic activities, and therefore the vascular protective and neuroprotective effects of bilirubin might contribute, in part, to the relationship between bilirubin and DPN in diabetic patients.

Our study has some limitations that need to be discussed. Firstly, we could not determine the causal relationships and provide a proven mechanism for the observed association between bilirubin and DPN due to the cross-sectional nature of our study. Secondly, our study population included only Chinese Han hospitalized patients with T2DM, and therefore our findings may not be generalizable to diabetic outpatients, other types of diabetes mellitus with different ethnicity, and non-diabetic population. Thirdly, all biochemical parameters including bilirubin, in most cases, were measured only once, which may underestimate the true association because of this variability over time. However, the use of standardized methods set in a single center, and measurements taken from subjects in a fasting state should improve reliability because there will be less fluctuation in TBIL levels than in the postprandial state. Fourthly, we chosen only WBC count and differential peripheral WBC as markers of inflammation because classic markers of inflammation such as CRP, TNF-ɑ, and IL-6 are not available. Lastly, although the well known risk factors were included in multivariable analysis to evaluate the association of serum TBIL with DPN, some residual or undetected confounding effects associated with serum TBIL cannot be ruled out. Despite these limitations, the strengths of the current study include its relatively large sample size and the very well-characterized nature of the patient cohort. Moreover, this study include the strict inclusion/exclusion criteria, and thorough multivariate adjustment.

## Conclusions

The present study showed that serum TBIL level significantly decreased in T2DM patients with DPN, and was an independent decisive factor for the presence of DPN. These findings suggest that lower physiological serum TBIL may be associated with the development of DPN. More well-designed prospective longitudinal studies are warranted to confirm our findings and further define the contribution of serum TBIL to the development of DPN.

## Data Availability

The data is available upon reasonable request to the corresponding author.

## Abbreviations

ALT: alanine aminotransferase
AST: aspartate aminotransferase
ANOVA: one-way analysis of variance
apoA: apolipoprotein A
apoB: apolipoprotein B
ApoB/ApoA: apolipoprotein B–apolipoprotein A ratio
ACR: albumin-to-creatinine ratio
APTT: activated partial thromboplastin time
ABI: ankle–brachial index
BMI: body mass index
CAN: cardiovascular autonomic neuropathy
CI: confidence intervals
CRP: C-reactive protein
CHD: coronary heart disease
Crea: creatinine
DPN: diabetic peripheral neuropathy
DSPN: distal symmetrical polyneuropathy
DBIL: direct bilirubin
DBP: diastolic blood pressure
DFU: diabetic foot ulceration
DN: diabetic nephropathy
DR: diabetic retinopathy
eNOS: endothelial nitric oxide synthase
eGFR: estimated glomerular filtration rate
FBG: fasting blood glucose
HDL-C: high-density lipoprotein cholesterol
HbA1c: glycated hemoglobin A1c
IL: interleukin
IBIL: indirect bilirubin
LDL-C: low-density lipoprotein cholesterol
MAP: mean arterial pressure
NLR: neutrophil to lymphocyte ratio
PTINR: international normalized ration
PP: pulse pressure
PT: prothrombin time
PAD: peripheral arterial disease
Q: quartile
ROC: receiver operating characteristic
ROS: reactive oxygen species
SD: standard deviation
SBP: systolic blood pressure
T2DM: type 2 diabetes mellitus
TNF-α: tumour necrosis factor alpha
TC: total cholesterol
TG: triglyceride
TBIL: total bilirubin
VPT: vibration perception threshold
WBC: white blood cell
